# Financial Resilience of Listed Hospital Companies in India Across Pre-COVID-19, COVID-19-Affected and Recovery Periods: A Longitudinal Ratio-Based Study

**DOI:** 10.7759/cureus.115536

**Published:** 2026-08-31

**Authors:** Arjavi Patel, Bhavinkumar Parmar

**Affiliations:** 1 Public Health, Independent Research, Ahmedabad, IND; 2 Artificial Intelligence, Generative AI, Machine Learning, Deep Learning, Healthcare, Digital Health, Natural Language Processing, Large Language Models, Health Informatics, Independent Research, New Jersey, USA

**Keywords:** covid-19, financial ratios, financial resilience, health economics, hospital finance, india, listed hospitals, market valuation

## Abstract

Background: COVID-19 created operational and financial pressure for hospitals. Financial resilience became important because hospitals require stable profitability, liquidity, debt-servicing capacity and market confidence to maintain services. Limited evidence is available on the financial resilience of listed hospital companies in India across pre-COVID-19, COVID-19-affected, and post-COVID-19 recovery periods. This study assessed period-wise differences in selected financial ratios, using a repeated-measures design that accounts for company-level clustering, to identify indicator-based evidence of financial resilience among selected listed hospital companies in India.

Materials and methods: This retrospective longitudinal secondary-data study used publicly available financial ratio data. Seven listed hospital companies with 10 years or more of comparable ratio data were included, out of an initial universe of 21 candidate companies. Final analysis covered 77 company-year observations from FY2015-16 to FY2025-26, classified as pre-COVID-19, COVID-19-affected, and post-COVID-19 recovery. Twenty-one non-redundant financial ratios covering profitability, liquidity, solvency, efficiency, market valuation, and per-share performance were analyzed (one originally planned outcome, market capitalization/net operating revenue, was excluded as mathematically identical to price/net operating revenue for a given company). Because the same seven companies were measured repeatedly across all three periods, each company's period-level mean was used as the unit of comparison, and the Friedman test (with company as a repeated-measures block) was used for period-wise comparison, with Wilcoxon signed-rank post hoc tests for significant ratios. Holm correction was applied across all 21 omnibus tests.

Results: The Friedman test showed significant period-wise differences in 5 of 21 financial ratios after Holm correction. Market valuation indicators (enterprise value (EV)/net operating revenue, price/net operating revenue) and per-share/earnings indicators (basic earnings per share (EPS), cash EPS, book value per share) showed significant improvement, concentrated mainly in the post-COVID-19 recovery period. Profitability ratios (profit before depreciation (PBDIT), profit before interest and tax (PBIT), profit before tax (PBT) and net profit margins, return on equity (ROE), return on capital employed (ROCE), return on assets (ROA)), liquidity ratios, interest coverage, debt-equity, asset turnover, EV/earnings before interest (EBITDA) and earnings yield did not show statistically significant period-wise differences once company-level clustering was properly accounted for.

Conclusions: Once repeated measurements from the same seven companies are treated as non-independent, listed hospital companies in India showed significant strengthening only in market valuation and per-share/earnings indicators during the post-COVID-19 recovery period, not in profitability or liquidity. Financial resilience, as measured through selected financial ratios, was concentrated in market-facing measures rather than operating performance. Findings should be interpreted as period-wise associations based on publicly available company-level data, not as causal effects of COVID-19, and should not be read as evidence about staffing, access, or clinical quality of care.

## Introduction

For the entire world, COVID-19 created a major shock for healthcare delivery and systems too. Hospitals had to manage infected patients, continue essential services, and adjust to sudden operational pressure. World Health Organization reports showed wide disruption in essential health services during the pandemic period [1]. Studies on health systems and their resilience also showed that countries needed capacity to absorb shocks, adapt service delivery, and maintain routine care during COVID-19 [2,3].

The pandemic's operational and financial pressures were not limited to the initial 2020 shock; continued viral evolution, including later variants such as Omicron sublineage JN.1, first characterized in the Indian setting [4], which became globally dominant in the fourth year of the pandemic, sustained infection-control costs, staffing pressure, and operational uncertainty well beyond the initial outbreak, which is part of the rationale for this study's multi-year "recovery" period rather than a single post-2020 endpoint.

Hospital resilience is not only clinical or operational. Financial capacity is also important because hospitals require regular cash flow, staff, supplies, equipment, infrastructure, and debt-servicing ability. Health financing studies during COVID-19 showed that resilient financing helps health systems absorb shocks and recover from emergency conditions [5]. A systematic review on hospitals during economic crisis also reported that hospitals are major users of health-system budgets and that efficiency with sustained performance becomes important during crisis periods [6].

For the purposes of this study, financial resilience is defined operationally as the extent to which a company's financial ratios - profitability, liquidity, debt-servicing capacity, and market valuation - are maintained or improved when comparing a defined "shock" period (COVID-19-affected) and a subsequent "recovery" period against a pre-shock baseline. This is a narrower, indicator-based definition than resilience frameworks used in health-systems research, which often incorporate operational continuity, staffing, and service delivery [2,3]. The present study does not measure those dimensions and makes no claim about them. Profitability, liquidity, and market valuation are treated as conceptually distinct constructs throughout this study rather than interchangeable proxies for "resilience," since a company can strengthen in one dimension without strengthening in another, a distinction directly relevant to our findings.

The financial health of hospitals has direct relevance for healthcare delivery. Hospitals with weak financial position may face difficulty in maintaining staffing, quality improvement, technology adoption, and service continuity. Evidence also suggests that hospital financial performance and quality of care are related, although this relationship may vary across settings and indicators [7]. One hospital finance study found that stronger hospital financial performance was associated with better quality and safety indicators in hospital care [8]. Therefore, financial performance is not only an accounting issue. It can influence hospital preparedness, service continuity, and long-term institutional stability.

COVID-19 affected hospital finances through several routes. Elective care declined in many settings, while expenses increased due to infection-control measures, staffing pressure, protective equipment, and changes in patient flow. Early pandemic evidence highlighted concern that financially insecure hospitals may have limited ability to invest in COVID-19 response and maintain staffing [9]. Empirical studies from the United States later showed mixed results. One study reported that COVID-19 relief funding helped offset operational losses in many US hospitals [10]. Another study also found that many hospitals had positive financial performance during 2020 and 2021, partly because of relief funds [11]. A study of large US teaching hospitals similarly found substantial variation in pandemic-period financial performance depending on hospital-specific factors [12].

Three recent studies offer close international comparators to the present analysis, each reaching different conclusions about the direction and uniformity of pandemic-period hospital financial performance. In the United States, Gidwani and Damberg [11] found that hospital operating margins reached all-time highs during 2020-2021 in a national cohort, but that improvement was uneven - hospitals serving Hispanic populations were more likely to experience new financial distress even after relief funding, indicating that aggregate improvement can mask distributional unevenness. In South Korea, Lee [13] used a linear mixed-effects model on 1,530 hospital-year observations and found that pandemic effects on financial performance differed systematically by hospital ownership, location, and type, a methodological point directly relevant here, since it demonstrates that hospital-level heterogeneity, not just period, drives financial outcomes, and that repeated-measures methods (as Lee used, and as we adopt in this study) are appropriate for this class of data. In China, Wang and Xu [14] compared private hospital profitability before and during COVID-19 and identified specific operational factors associated with profitability change, rather than assuming a uniform sector-wide effect. Taken together, these three studies caution against treating "hospital financial resilience" as a single, uniform phenomenon: national context, funding structure, hospital type, and the statistical treatment of repeated measurements all shape the conclusions drawn. The present study's revised analytic approach, treating company as a repeated-measures unit rather than pooling company-years as independent, follows directly from this literature, particularly Lee [13].

Indian healthcare system has a large private-sector presence. India has a mixed public-private healthcare structure that serves a large and diverse population [15]. During COVID-19, private healthcare capacity was considered important for pandemic response, but its use and integration varied across settings [16]. Financial viability of private hospitals also remains a current policy issue in India, especially in relation to reimbursement systems and health insurance schemes [17]. This makes the financial performance of Indian private hospital companies an important area for study.

Earlier Indian evidence also supports the use of financial-ratio analysis for private hospitals. An earlier Indian study examined the financial health of private sector hospitals in India using balance sheet and profit and loss data and identified several dimensions of financial health through financial ratios [18]. However, limited evidence is available on how listed hospital companies in India performed across pre-COVID-19, COVID-19-affected, and post-COVID-19 recovery periods. Most available discussions focus on health-system pressure, service disruption, or hospital operations. Less attention has been given to listed hospital companies as corporate healthcare providers.

This study addresses this gap by assessing the ratio-based financial resilience of selected listed hospital companies in India during pre-COVID-19, COVID-19-affected, and post-COVID-19 recovery periods. Financial resilience was examined through profitability, liquidity, solvency, efficiency, market valuation, and per-share indicators. This period-wise analysis helps understand whether recovery-period performance differed from the pre-COVID-19 baseline and whether improvement was uniform across financial dimensions.

Research questions

This study attempted to explore answers to the following research questions:

RQ1. Which financial ratios showed significant period-wise differences across pre-COVID-19, COVID-19-affected, and post-COVID-19 recovery periods?

RQ2. Which financial ratios showed significant improvement during the post-COVID-19 recovery period compared with the pre-COVID-19 baseline?

## Materials and methods

Study design

This study used a retrospective longitudinal secondary-data design to examine the financial resilience of selected listed hospital companies in India across pre-COVID-19, COVID-19-affected, and post-COVID-19 recovery periods. This design was suitable because the study used already available company-level financial ratio data and compared the same sector across multiple financial years.

Data source

Financial ratio data were collected from the Moneycontrol website, a publicly accessible financial information platform [19]. Financial ratios included in this study were based on consolidated ratios. Data were extracted in May 2026, consistent with the market-capitalization eligibility snapshot used for sample selection. The database contained ratio information for selected listed hospital companies in India. Company-year observations were used as the unit of analysis. Financial ratios were considered suitable because ratio analysis standardizes financial statement information and allows comparison across companies and time periods despite differences in firm size [20-22].

Sample selection

This study included selected listed hospital companies in India for which 10 years or more of comparable ratio data were available in the source database. Companies were included only when sufficient longitudinal ratio data were available to classify observations into pre-COVID-19, COVID-19-affected, and post-COVID-19 recovery periods. The final sample consisted of seven listed hospital companies observed over eleven financial years from FY2015-16 to FY2025-26. This yielded 77 company-year observations.

Seven companies included in the study were Apollo Hospitals Enterprise Ltd., Fortis Healthcare Ltd., Indraprastha Medical Corporation Ltd., Kovai Medical Center and Hospital Ltd., KMC Speciality Hospitals (India) Ltd., Narayana Hrudayalaya Ltd. and Healthcare Global Enterprises Ltd.

The inclusion criterion was the availability of at least 10 years of financial ratio data for a listed hospital company. Companies without sufficient longitudinal ratio data or without comparable ratio information across the required study period were excluded. This criterion was used to maintain comparability across firms and support period-wise analysis. The initial study universe had 21 hospital companies listed on BSE or NSE with market capitalization more than ₹1,000 crore as of May 2026. Ten companies were listed for less than five years. Four of them were listed for five to less than 10 years. These 14 companies were excluded. A minimum listing period of more than 10 years was required to obtain sufficient and comparable financial ratio observations across pre-COVID-19, COVID-19-affected, and post-COVID-19 recovery periods. Thus, seven hospitals were included in this study. This exclusion criterion was based solely on listing duration and data availability, not on financial performance; the potential for survivorship bias this introduces is discussed in the Limitations. Initial study universe had 21 hospital companies listed on BSE or NSE with market capitalization more than ₹1,000 crore as of May 2026 (Table 1).

**Table 1:** Participant flow from initial universe to final sample

Step	Companies excluded	Companies remaining
Initial universe: listed hospital companies (BSE/NSE), market cap > ₹1,000 crore as of May 2026	—	21
Excluded: listed < 5 years	10	11
Excluded: listed 5 to <10 years	4	7
Final sample (≥10 years of comparable ratio data, FY2015-16 to FY2025-26)	—	7

Period classification

The study period was divided into three phases. FY2015-16 to FY2019-20 was classified as the pre-COVID-19 period. FY2020-21 to FY2021-22 was classified as the COVID-19-affected period. FY2022-23 to FY2025-26 was classified as the post-COVID-19 recovery period.

This classification allowed comparison of financial performance before the pandemic shock, during the affected period, and during the recovery period. The final dataset contained 35 company-year observations in the pre-COVID-19 period, 14 observations in the COVID-19-affected period, and 28 observations in the post-COVID-19 recovery period. Table 2 summarizes the study period classification and number of observations.

**Table 2:** Study period classification and observations COVID-19: coronavirus disease 2019; FY: financial year.

Period	Financial years	Number of years	Company-year observations	Companies represented
Pre-COVID-19	FY2015-16 to FY2019-20	5	35	7
COVID-19-affected	FY2020-21 to FY2021-22	2	14	7
Post-COVID-19 recovery	FY2022-23 to FY2025-26	4	28	7
Total	FY2015-16 to FY2025-26	11	77	7

Variables and measures

Financial resilience was assessed through 22 financial ratios covering profitability, liquidity, solvency and debt-servicing ability, efficiency, market valuation and per-share performance. Table 3 defines the selected financial ratios used in the present study.

**Table 3:** Financial dimensions and selected ratios PBDIT: profit before depreciation, interest and tax; PBIT: profit before interest and tax; PBT: profit before tax; ROE: return on equity; ROCE: return on capital employed; ROA: return on assets; EV: enterprise value; EBITDA: earnings before interest, tax, depreciation and amortization; EPS: earnings per share; BV: book value; X: times or multiple; Rs.: Indian rupees.

Dimension	Ratio	Definition	Direction of interpretation
Profitability	PBDIT margin	Profit before depreciation, interest and tax divided by net operating revenue, expressed as a percentage.	Higher value indicates stronger operating surplus before depreciation and finance cost.
Profitability	PBIT margin	Profit before interest and tax divided by net operating revenue, expressed as a percentage.	Higher value indicates stronger operating profit after depreciation but before finance cost.
Profitability	PBT margin	Profit before tax divided by net operating revenue, expressed as a percentage.	Higher value indicates stronger pre-tax profitability.
Profitability	Net profit margin	Net profit divided by net operating revenue, expressed as a percentage.	Higher value indicates stronger bottom-line profitability.
Profitability	Return on net worth/equity	Net profit relative to shareholders' equity or net worth, expressed as a percentage.	Higher value indicates stronger return to equity shareholders.
Profitability	Return on capital employed	Operating profit relative to capital employed, expressed as a percentage.	Higher value indicates better return on long-term capital used in the business.
Profitability	Return on assets	Net profit relative to total assets, expressed as a percentage.	Higher value indicates more effective use of assets for generating profit.
Liquidity	Current ratio	Current assets divided by current liabilities.	Higher value indicates stronger short-term liquidity.
Liquidity	Quick ratio	Liquid current assets divided by current liabilities.	Higher value indicates stronger immediate liquidity after excluding less liquid current assets.
Solvency and debt servicing	Total debt/equity	Total debt divided by shareholders' equity or net worth.	Lower value generally indicates lower financial leverage.
Solvency and debt servicing	Interest coverage ratio	Earnings available for interest payment divided by interest expense.	Higher value indicates stronger ability to service interest obligations.
Efficiency	Asset turnover ratio	Net operating revenue divided by total assets.	Higher value indicates more efficient use of asset base for generating revenue.
Market valuation	EV/net operating revenue	Enterprise value divided by net operating revenue.	Higher value indicates stronger revenue-based market valuation.
Market valuation	EV/EBITDA	Enterprise value divided by earnings before interest, tax, depreciation and amortization.	Higher value indicates higher market valuation relative to operating cash earnings.
Market valuation	Market capitalization/net operating revenue	Market capitalization divided by net operating revenue.	Higher value indicates stronger equity-market valuation relative to revenue.
Market valuation	Price/book value	Market price per share divided by book value per share.	Higher value indicates stronger market valuation relative to accounting book value.
Market valuation	Price/net operating revenue	Market price per share divided by net operating revenue per share.	Higher value indicates stronger price-to-sales valuation.
Market valuation	Earnings yield	Earnings per share divided by market price per share.	Higher value indicates higher earnings generated per unit of market price.
Per-share performance	Basic earnings per share	Profit attributable to equity shareholders divided by the weighted average number of equity shares.	Higher value indicates stronger earnings attributable to each share.
Per-share performance	Cash earnings per share	Cash profit divided by number of equity shares.	Higher value indicates stronger cash earnings attributable to each share.
Per-share performance	Revenue from operations per share	Revenue from operations divided by the number of equity shares.	Higher value indicates stronger operating revenue generated per share.
Per-share performance	Book value excluding revaluation reserve per share	Shareholders' funds excluding revaluation reserve divided by number of equity shares.	Higher value indicates stronger accounting net worth per share excluding revaluation reserve.

Market capitalization/net operating revenue was subsequently excluded from the analysis (see Statistical analysis) after confirming it is mathematically identical to price/net operating revenue for a given company (Market Cap = Price × Shares Outstanding; Net Operating Revenue/Shares = Net Operating Revenue per Share; therefore Market Cap/Net Operating Revenue = Price/Net Operating Revenue per Share). This was confirmed empirically: correlation = 1.00 across all 77 company-year observations in this dataset. Retaining both as separate outcomes would have double-counted a single underlying signal. The final analysis therefore used 21 non-redundant ratios. Inventory turnover ratio was excluded from the final analysis. The source database reported zero values for several company-year observations. This ratio also had limited conceptual relevance for hospital service companies. Asset turnover ratio was retained as an efficiency measure because it better reflects the ability of hospital companies to use the asset base to generate operating revenue.

Statistical analysis

Data were analyzed using IBM SPSS Statistics for Windows, Version 27 (Released 2019; IBM Corp., Armonk, New York, United States) and Python (SciPy). Descriptive statistics were computed for all selected financial ratios. The dataset contained no missing values across any of the 21 analyzed ratios for any company-year observation, so no missing-data imputation or exclusion rule was required. No extreme outliers requiring special treatment (e.g., winsorization or exclusion) were identified in the ratio data; all reported values reflect the ratios as sourced. Because the 77 company-year observations represent repeated measurements from only seven companies rather than 77 independent units, company-year observations were first aggregated to a company-by-period mean for each of the 21 non-redundant ratios, yielding one value per company per period (7 companies × 3 periods). Normality was not assumed. Because each company contributed one paired observation per period, the Friedman test [23] - the non-parametric analog of repeated-measures ANOVA, appropriate when the same subjects (here, companies) are measured under multiple related conditions (here, periods) - was used for the omnibus period-wise comparison for each ratio, with company as the repeated-measures block. This approach directly addresses the non-independence of company-year observations, since the unit of statistical inference is the company (n = 7), not the company-year (n = 77).

For ratios with a significant omnibus Friedman result, Wilcoxon signed-rank tests [24] (paired by company) were used for pairwise post hoc comparisons between periods. Given the small number of independent units (seven companies), exact Wilcoxon signed-rank p-values were computed. Holm's sequential Bonferroni procedure [25] was applied to control the family-wise error rate across all 21 omnibus tests, and separately within each ratio's pairwise comparisons, rather than applying a simple Bonferroni correction only within post hoc comparisons. Statistical significance was assessed at Holm-adjusted p < .05.

Primary outcomes were not formally prespecified before data collection; this is acknowledged as a limitation. All 21 ratios were treated as co-primary and subjected to the same correction. No stock splits, bonus share issuances, or share buybacks occurred for any of the seven included companies during the study period (FY2015-16 to FY2025-26). One rights issue fell within this window: Healthcare Global Enterprises Ltd. completed a 1:17 rights issue, with shares allotted on 27 March 2026 and listed on 1 April 2026, within the final week of FY2025-26. This increased outstanding shares by approximately 5.88% for roughly five days of the financial year, an estimated effect of less than 0.1% on the annual weighted-average share denominator used for basic EPS, with a correspondingly marginal effect on book value per share; per-share indicators were therefore not separately adjusted for this event. Mergers and acquisitions occurring among the included companies during the study period were not adjusted for in the ratio calculations, consistent with standard practice in descriptive, retrospective secondary-data studies; both points are noted as limitations below. Reported per-share values are in nominal terms and were not adjusted for inflation; this is noted as a limitation. No a priori power calculation was performed. This study used all companies meeting the eligibility criteria (Table 1) rather than a pre-planned sample size targeting a specific statistical power, consistent with standard practice for retrospective secondary-data studies; a retrospective power calculation was not considered advisable and was not performed.

Ethical considerations

Ethical approval was not required because this study used publicly available company-level secondary data. No human participants, patient records, identifiable information, clinical data, animal data, or institutional medical records were used. Analysis was limited to financial ratio information of listed companies available in the public domain.

Data availability

The financial ratio dataset (company-year observations for all 21 analyzed ratios) and the analysis code (Python/SciPy) used to generate the results in this manuscript are available from the corresponding author on reasonable request.

## Results

Period-wise comparison of financial ratios (repeated-measures analysis)

Because company-year observations are repeated measurements from seven companies, each company's period-level mean was used as the unit of analysis, and the Friedman test was used for omnibus period-wise comparison, with Holm correction applied across all 21 outcomes. Table 4 presents Friedman test results.

**Table 4:** Friedman test results for period-wise differences (Holm-corrected across 21 outcomes) PBDIT: profit before depreciation, interest and tax; PBIT: profit before interest and tax; PBT: profit before tax; EV: enterprise value; EPS: earnings per share; BV: book value; X: times or multiple

Ratio	Pre-COVID-19 median	COVID-19 median	Recovery median	χ²(df=2)	p (raw)	p (Holm)	Decision
EV/Net Operating Revenue (X)	2.39	3.80	4.45	14.00	< .001	0.019	Significant
Price/Net Operating Revenue (X)	2.04	3.67	4.28	14.00	< .001	0.019	Significant
Cash EPS (Rs.)	7.30	8.31	19.69	12.29	0.002	0.041	Significant
Basic EPS (Rs.)	3.14	3.32	15.13	12.29	0.002	0.041	Significant
Book Value Excl. Reval./Share (Rs.)	56.19	63.87	117.45	12.29	0.002	0.041	Significant
PBIT Margin (%)	7.06	8.55	15.55	11.14	0.004	0.061	Not significant
Quick Ratio (X)	0.81	1.34	1.27	11.14	0.004	0.061	Not significant
Revenue from Operations/Share (Rs.)	96.16	90.70	147.12	10.57	0.005	0.071	Not significant
PBDIT Margin (%)	11.98	15.18	20.51	10.29	0.006	0.076	Not significant
Current Ratio (X)	0.94	1.41	1.34	8.86	0.012	0.143	Not significant
PBT Margin (%)	4.89	6.42	14.13	8.86	0.012	0.143	Not significant
Net Profit Margin (%)	3.20	4.60	10.51	8.86	0.012	0.143	Not significant
Interest Coverage Ratio (X)	3.14	3.98	8.44	8.86	0.012	0.143	Not significant
Asset Turnover Ratio (X)	0.95	0.74	0.91	6.00	0.050	0.398	Not significant
Return on Assets (%)	2.95	5.21	10.99	6.00	0.050	0.398	Not significant
Return on Capital Employed (%)	7.46	11.38	19.39	5.43	0.066	0.398	Not significant
Price/BV (X)	3.87	3.88	5.99	4.57	0.102	0.509	Not significant
Return on Net Worth/Equity (%)	7.61	10.85	18.52	3.43	0.180	0.720	Not significant
Total Debt/Equity (X)	0.48	0.42	0.43	3.19	0.203	0.720	Not significant
Earnings Yield	0.014	0.010	0.028	2.30	0.317	0.720	Not significant
EV/EBITDA (X)	17.66	18.69	20.03	2.00	0.368	0.720	Not significant

Five of 21 ratios (24%) remained statistically significant after Holm correction: EV/net operating revenue, price/net operating revenue, cash EPS, basic EPS, and book value excluding revaluation reserve per share. Notably, no profitability ratio (PBDIT, PBIT, PBT, or net profit margin; ROE, ROCE, ROA) reached significance once company-level clustering was properly accounted for, despite showing an apparent upward pattern across periods. Liquidity ratios (current ratio, quick ratio), interest coverage, debt-equity, asset turnover, EV/EBITDA, and earnings yield also did not show statistically significant period-wise differences.

Post hoc pairwise comparisons

For ratios with a significant omnibus result, Wilcoxon signed-rank tests (paired by company) were used to identify which specific periods differed, with Holm correction applied within each ratio's three pairwise comparisons. Table 5 presents Wilcoxon signed-rank post hoc comparison results.

**Table 5:** Wilcoxon signed-rank post hoc comparisons for significant ratios (Holm-corrected within each ratio) EV: enterprise value; EPS: earnings per share

Ratio	Comparison	p (raw)	p (Holm)	Decision
EV/Net Operating Revenue	Pre-COVID-19 vs COVID-19	0.016	0.047	Significant
EV/Net Operating Revenue	Pre-COVID-19 vs Recovery	0.016	0.047	Significant
EV/Net Operating Revenue	COVID-19 vs Recovery	0.016	0.047	Significant
Price/Net Operating Revenue	Pre-COVID-19 vs COVID-19	0.016	0.047	Significant
Price/Net Operating Revenue	Pre-COVID-19 vs Recovery	0.016	0.047	Significant
Price/Net Operating Revenue	COVID-19 vs Recovery	0.016	0.047	Significant
Cash EPS	Pre-COVID-19 vs COVID-19	0.078	0.078	Not significant
Cash EPS	Pre-COVID-19 vs Recovery	0.016	0.047	Significant
Cash EPS	COVID-19 vs Recovery	0.016	0.047	Significant
Basic EPS	Pre-COVID-19 vs COVID-19	0.109	0.109	Not significant
Basic EPS	Pre-COVID-19 vs Recovery	0.016	0.047	Significant
Basic EPS	COVID-19 vs Recovery	0.016	0.047	Significant
Book Value Excl. Reval./Share	Pre-COVID-19 vs COVID-19	0.109	0.109	Not significant
Book Value Excl. Reval./Share	Pre-COVID-19 vs Recovery	0.016	0.047	Significant
Book Value Excl. Reval./Share	COVID-19 vs Recovery	0.016	0.047	Significant

EV/net operating revenue and price/net operating revenue (mathematically linked; see Methods) increased progressively across all three periods, with every pairwise comparison significant. Cash EPS, basic EPS, and book value per share followed a different pattern: no significant difference between pre-COVID-19 and COVID-19-affected periods, but significant increases by the recovery period, indicating that per-share/earnings improvement was concentrated in the recovery period rather than emerging during the COVID-19-affected years themselves.

Hodges-Lehmann estimates and 95% confidence intervals for each pairwise comparison (Table 6) confirm the direction and magnitude of these differences; confidence intervals excluding zero align with statistically significant Holm-adjusted comparisons, while the three non-significant comparisons (cash EPS, basic EPS, and book value per share, each pre-COVID-19 vs COVID-19) have confidence intervals spanning zero.

**Table 6:** Hodges-Lehmann estimates and 95% confidence intervals for significant pairwise comparisons EV: enterprise value; EPS: earnings per share

Ratio	Comparison	HL Estimate	95% CI
EV/Net Operating Revenue	Pre-COVID-19 vs COVID-19	0.98	0.08 to 1.83
EV/Net Operating Revenue	Pre-COVID-19 vs Recovery	1.73	0.76 to 2.70
EV/Net Operating Revenue	COVID-19 vs Recovery	0.78	0.19 to 1.23
Price/Net Operating Revenue	Pre-COVID-19 vs COVID-19	1.07	0.16 to 1.99
Price/Net Operating Revenue	Pre-COVID-19 vs Recovery	1.84	0.99 to 2.79
Price/Net Operating Revenue	COVID-19 vs Recovery	0.81	0.25 to 1.40
Cash EPS	Pre-COVID-19 vs COVID-19	9.12	−0.89 to 41.54
Cash EPS	Pre-COVID-19 vs Recovery	35.37	7.60 to 106.95
Cash EPS	COVID-19 vs Recovery	26.45	6.41 to 70.10
Basic EPS	Pre-COVID-19 vs COVID-19	5.38	−2.14 to 23.88
Basic EPS	Pre-COVID-19 vs Recovery	28.29	3.91 to 73.70
Basic EPS	COVID-19 vs Recovery	22.01	4.84 to 58.35
Book Value Excl. Reval./Share	Pre-COVID-19 vs COVID-19	12.13	−3.75 to 127.93
Book Value Excl. Reval./Share	Pre-COVID-19 vs Recovery	91.95	11.37 to 379.30
Book Value Excl. Reval./Share	COVID-19 vs Recovery	76.68	8.86 to 251.38

Company-specific trajectories

Company-level heterogeneity was evident even among the five significant ratios (Figure 1). The improvement in per-share/earnings measures (basic EPS, cash EPS, book value per share) was driven disproportionately by two companies (Kovai Medical Center and Hospital and Apollo Hospitals Enterprise) while the remaining five companies (Fortis Healthcare, Indraprastha Medical Corporation, KMC Speciality Hospitals, Narayana Hrudayalaya, and Healthcare Global Enterprises) showed comparatively flat trajectories near the lower end of the range across the full study period. This indicates the significant omnibus result for these indicators reflects a company-concentrated pattern rather than a uniform improvement across all seven companies and should be interpreted accordingly.

**Figure 1:**
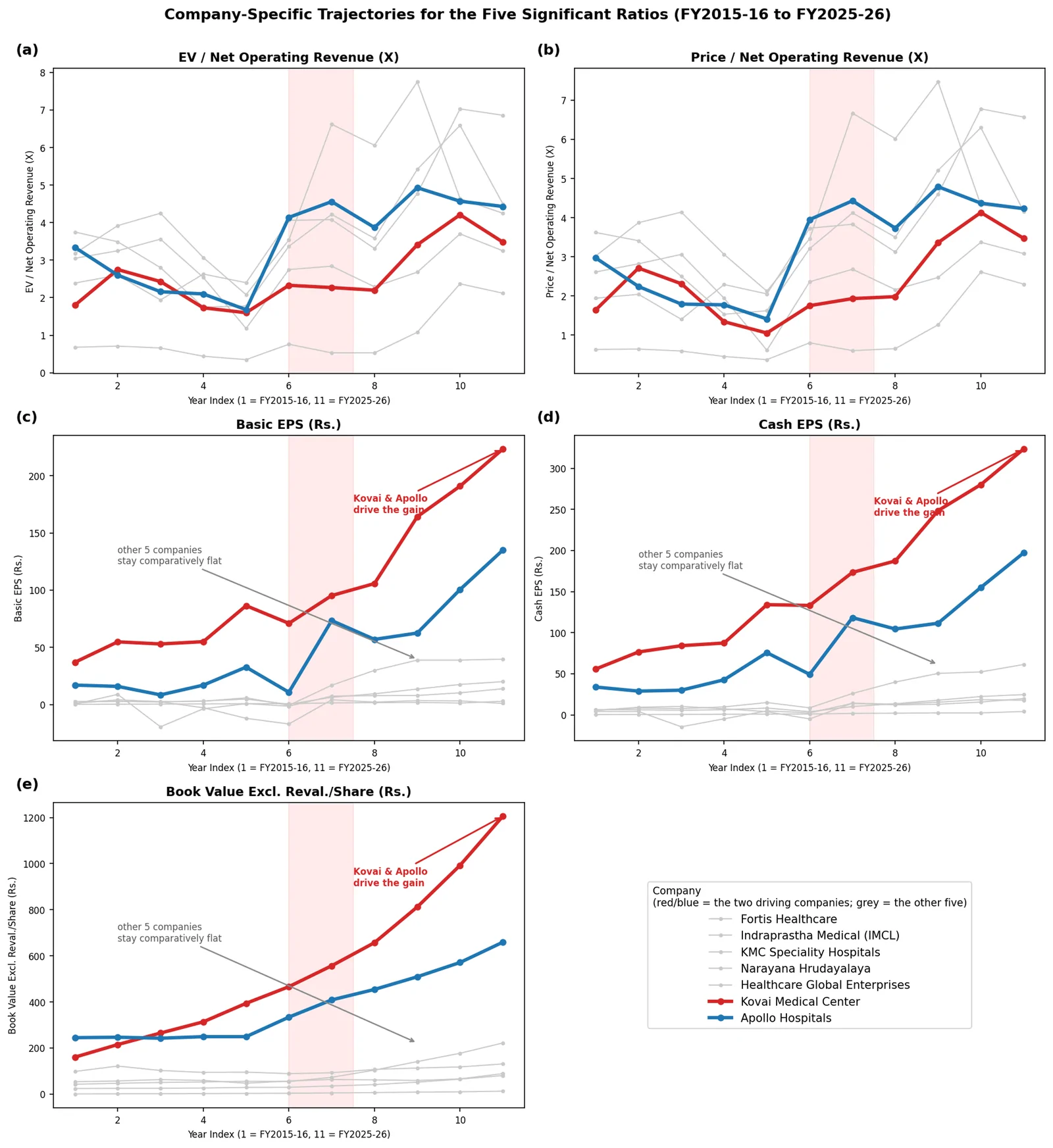
Company-specific trajectories for the five significant ratios (FY2015-16 to FY2025-26). (a) EV/net operating revenue, (b) price/net operating revenue, (c) basic EPS, (d) cash EPS, and (e) book value excluding revaluation reserve per share. Red and blue lines indicate the two companies (Kovai Medical Center and Apollo Hospitals, respectively) driving most of the observed gains; grey lines indicate the remaining five companies. EV: enterprise value; EPS: earnings per share

Summary

After correcting for company-level clustering and applying Holm correction across 21 non-redundant outcomes, 5 of 21 financial ratios showed statistically significant period-wise differences - market valuation (EV/net operating revenue, price/net operating revenue) and per-share/earnings measures (basic EPS, cash EPS, book value per share). No profitability or liquidity ratio remained significant. This is a materially different conclusion from the original analysis, which found 17 of 22 ratios significant including all profitability indicators; that analysis is now understood to have been anti-conservative due to treating repeated company-year measurements as independent.

## Discussion

This study assessed the financial resilience of selected listed hospital companies in India across pre-COVID-19, COVID-19-affected, and post-COVID-19 recovery periods, using a repeated-measures analysis that treats each company as its own block across periods - addressing a methodological limitation (non-independence of company-year observations) identified in peer review. After Holm correction across 21 non-redundant ratios, only five showed statistically significant period-wise differences, and none were profitability or liquidity measures.

This is a substantially narrower finding than an initial, uncorrected analysis suggested, and the difference is instructive. Market valuation indicators (EV/net operating revenue, price/net operating revenue) and per-share/earnings measures (basic EPS, cash EPS, book value per share) improved significantly, concentrated mainly in the post-COVID-19 recovery period. These are market-facing measures, reflecting investor pricing and per-share accounting outcomes, not direct measures of operating profitability or short-term liquidity. Profitability ratios showed a visually similar upward pattern across periods (medians in Table 4), but this pattern did not reach statistical significance once the seven companies were treated as the unit of inference rather than the 77 company-years - a result consistent with the possibility that the original apparent profitability improvement reflected a small number of companies or years driving the pattern, rather than a broad, consistent sector-wide shift.

Comparison with international evidence

These findings sit alongside three closely comparable international studies, each of which found effects that were uneven rather than uniform. In the United States, Gidwani and Damberg [11] found record aggregate operating margins during 2020-2021 but substantial distributional unevenness, with hospitals serving Hispanic populations disproportionately likely to experience new financial distress despite relief funding, indicating that period-level aggregates can mask company- or population-level heterogeneity. In South Korea, Lee [13], using a linear mixed-effects model appropriate for repeated hospital-level measurements, found that pandemic financial effects differed systematically by ownership, location, and hospital type; this methodological choice directly parallels the repeated-measures approach adopted in this revision and reinforces that hospital-level clustering materially affects conclusions in this literature. In China, Wang and Xu [14] found that private hospital profitability changes before and during COVID-19 were associated with specific operational factors rather than a uniform pandemic effect. Read together with the present findings - where profitability differences were not statistically robust once clustering was addressed, while valuation and per-share measures were - this body of evidence argues against treating "hospital financial resilience" during COVID-19 as a single, uniform, or purely period-driven phenomenon in any country studied so far, including India.

The pandemic's disruption was not confined to a single defined window; continued viral evolution, including variants such as JN.1, documented in the Indian setting [4], that became globally dominant well into the pandemic's later years, is one plausible reason financial effects continued to evolve across the study's multi-year "recovery" period rather than resolving immediately after the initial 2020-2021 shock.

Interpretation and clinical relevance

These findings should be interpreted narrowly. Stronger market valuation and per-share metrics indicate that investors priced these companies more favorably and that reported per-share earnings and book value rose during the recovery period; they do not demonstrate improved staffing, access to care, clinical preparedness, or quality of care, and this study makes no claim in that direction. Prior evidence on the relationship between hospital financial performance and quality of care is itself mixed and setting-dependent [7,8]. Extending market-valuation findings from listed hospital companies to inferences about care delivery would not be supported by this study's design or data. Any association between financial indicators and hospital resilience in a clinical or operational sense would require patient-level, staffing, or service-continuity data that were not available here.

Several factors besides COVID-19 itself may explain the observed patterns and should temper causal interpretation: general economic and market growth over an eleven-year window, company-specific acquisitions, expansion, or debt restructuring, inflation, changes in health-insurance reimbursement, and broader capital-market movements unrelated to hospital operations specifically. Market valuation ratios in particular are influenced by sector-wide investor sentiment and capital-market conditions rather than being pure indicators of hospital operating performance.

Limitations

This study has several limitations beyond those already discussed. Analysis is limited to seven listed hospital companies identified from an initial universe of 21 candidates meeting a market-capitalization threshold (Table 1); companies excluded for having a shorter listing history may differ systematically from those retained, introducing potential survivorship bias that this study cannot rule out. A sensitivity analysis using alternative listing-duration thresholds (e.g., including companies with 5-10 years of listing history) was not possible, as financial ratio data were not collected for companies excluded during sample selection; this remains a limitation of the present analysis. With only seven independent clusters, the repeated-measures tests used here have limited statistical power, and null results for profitability and liquidity ratios should not be over-interpreted as evidence of no effect; they indicate the effect, if present, was not detectable with this sample size and this level of correction for multiple comparisons. Primary outcomes were not prespecified before data collection. Financial resilience was assessed indirectly through selected financial ratios rather than through a validated composite resilience construct; profitability, liquidity, and market valuation are conceptually distinct and were examined separately rather than combined into a single index, consistent with the operational definition set out in the Introduction. A rights issue by one included company (Healthcare Global Enterprises Ltd., 1:17, shares listed 1 April 2026) fell within the final days of the study period; its estimated effect on per-share metrics was negligible (<0.1% on the weighted-average share count) and was not separately modeled. Mergers and acquisitions occurring among the included companies during the study period were also not adjusted for in the ratio calculations; while this is standard practice for descriptive, retrospective secondary-data analyses, it means reported ratios reflect each company's as-reported financials without correction for the effect of corporate restructuring on comparability across years. Future research combining financial ratios with hospital capacity, occupancy, and quality indicators, using panel or mixed-effects methods with a larger number of clustering units, would provide a more complete assessment of financial and operational resilience.

## Conclusions

Once repeated company-year measurements are properly treated as non-independent, listed hospital companies in India showed statistically significant strengthening only in market valuation (EV/net operating revenue, price/net operating revenue) and per-share/earnings indicators (basic EPS, cash EPS, book value per share) during the post-COVID-19 recovery period, concentrated mainly in the recovery period rather than the COVID-19-affected years. Profitability and liquidity ratios did not show statistically robust period-wise differences after correcting for company-level clustering and multiple comparisons, despite showing an uncorrected upward pattern. Financial resilience, as measured through selected financial ratios, was therefore concentrated in market-facing measures rather than operating performance in this sample. Findings should be interpreted as period-wise associations based on publicly available company-level data from seven companies, not as causal effects of COVID-19, and should not be extended to claims about staffing, access, or clinical quality of care.
